# Top management team faultlines and corporate industrial diversification: The mediating role of strategic attentional breadth

**DOI:** 10.3389/fpsyg.2023.1102192

**Published:** 2023-02-23

**Authors:** Weihong Chen, Chen Chen, Xiaoguo Xiong

**Affiliations:** ^1^School of Economics, Guangxi University, Nanning, China; ^2^School of Business Administration, Guangxi University, Nanning, China

**Keywords:** task-related TMT faultlines, relationship-related TMT faultlines, industrial diversification, strategic attentional breadth, China

## Abstract

**Introduction:**

Integrating faultline theory and the attention-based view, this paper explores the impact and process mechanisms of two types of faultlines (i.e., task-related faultlines and relationship-related faultlines) within top management teams (TMTs), specifically on corporate industrial diversification.

**Methods:**

Based on the unbalanced panel data of Chinese A-share non-financial listed firms from 2008-2021, this study uses the fixed-effects model for hypothesis testing.

**Results:**

First, task-related TMT faultlines promote corporate industrial diversification, while conversely, relationship-related TMT faultlines inhibit corporate industrial diversification. Second, task-related TMT faultlines promote firms’ strategic attentional breadth, while conversely, relationship-related TMT faultlines inhibit firms’ strategic attentional breadth. Third, strategic attentional breadth plays a partially-mediating role in the relationship between both types of TMT faultlines and firms’ industrial diversification.

**Discussion:**

This study extends the research related to corporate industrial diversification based on micro-level explanatory mechanisms, and also provides implications and guidance for the rational allocation of TMT and firms’ industrial diversification management practices.

## 1. Introduction

Industrial diversification (also known as inter-industrial diversification), i.e., expansion into businesses new to the firm (Rumelt, 1982; Hitt et al., 1997), has been the main business form adopted by most firms in developed economies from the 1950s and 1960s until now (Mayer et al., 2017; Mendoza-Abarca and Gras, 2019). Most firms in emerging economies (Batsakis and Mohr, 2017; Gyan et al., 2017; Jiao et al., 2020) have also adopted this form in recent years. Determining whether or not to implement industrial diversification – and deciding on the extent of industrial diversification – are two of the most challenging and critical decisions faced by firm managers (Schommer et al., 2019; Guerras-Martin et al., 2020; Choi et al., 2021). With the continuous development of technological innovation, industrial integration and the digital economy, the industrial diversification boom characterized by “cross-border business” has been ushered in. In addition, industrial diversification has invoked a new connotation in modern times, while also facing new challenges. This has given rise to a question of great theoretical and practical value. Specifically, in an era of volatility, uncertainty, complexity and ambiguity, what factors affect corporate industrial diversification?

A TMT plays a key role in the formulation and implementation of a firm’s corporate strategic decisions and has a profound impact on the development and future business performance of that firm. Previous studies have extensively discussed the impact of TMT characteristics on industrial diversification, such as TMT members’ ages (Wiersema and Bantel, 1992), TMT members’ tenure (Wiersema and Bantel, 1992; Boeker, 1997), TMT members’ functional background (Song, 1982; Boeker, 1997), and TMT members’ educational backgrounds (Wiersema and Bantel, 1992; Patzelt et al., 2009). Further, some scholars have discussed the impact of TMT structural characteristics on industrial diversification. Examples include TMT age heterogeneity (Wiersema and Bantel, 1992), TMT education level heterogeneity (Wiersema and Bantel, 1992; Mehrabi et al., 2021), TMT tenure heterogeneity (Boeker, 1997; Mehrabi et al., 2021), and TMT functional background heterogeneity (Sidhu et al., 2020; Mehrabi et al., 2021). However, existing literature on TMT structural characteristics has focused on a single characteristic-based index of heterogeneity (Jackson et al., 2003). Such studies ignore the possible existence of subgroups within a TMT (Richard et al., 2019). Specifically, TMT members have multiple attributes and are divided into subgroups based on differences in these attributes (Carton and Cummings, 2012). In turn, these different attributes affect the process of strategic decision making and implementation, and eventually affect team outcomes (Hutzschenreuter and Horstkotte, 2013; Li and Jones, 2019; Ma et al., 2021). Studies have shown that TMT faultlines have a significant impact on strategic decisions, such as those made in relation to green technology innovation (Ma et al., 2021), over-investment (Xie et al., 2022), strategic change (Richard et al., 2019; Wu et al., 2021; Zhang et al., 2021), service transition (Chen and Wang, 2021), competitive behavior (Li and Jones, 2019), and entrepreneurial performance (Qiu et al., 2022). In light of the significant explanatory power of TMT faultlines, this paper suggests that linking TMT faultlines and corporate industrial diversification is a worthwhile research endeavor, as the industrial diversification literature in recent years has increasingly focused on the important role of top managers’ representations and perceptions (Neely et al., 2020; Elia et al., 2021). Therefore, this paper employs faultline theory to explore the differential effects of different types of TMT faultlines on corporate industrial diversification.

In addition, previous studies exploring TMT demographic characteristics (or structural characteristics) and corporate industrial diversification have placed very little focus on the process mechanisms by which TMT characteristics impact industrial diversification. From a more cognitive perspective, Barr et al. (1992) argued that, after environmental changes, top managers’ mental models change in response to adaptive strategy changes. The authors also argued that managers’ mental models are better predictors than managers’ characteristics in terms of whether or not their business strategy will change. This view was echoed in Cho and Hambrick’s (2006) study, which clearly suggested that it is TMT characteristics that greatly influence the likelihood of mental models. Based on the case of airline deregulation, the study showed that strategic attention is a mediating factor between TMT characteristics and firms’ strategic changes. In order to open the “black box” between TMT faultlines and corporate industrial diversification (Neely et al., 2020), this paper intends to integrate faultline theory and the attention-based view to further explore the impact of two types of TMT faultlines on strategic attentional breadth. In addition, the mediating role of strategic attentional breadth in the relationship between two types of TMT faultlines and corporate industrial diversification is examined.

The contribution of this paper is in two main aspects. Firstly, based on faultline theory, this paper provides a new explanation for corporate diversification from the perspective of a TMT’s internal structural characteristics. Previous studies have extensively explored the important impact of TMT characteristics on corporate industrial diversification. However, these studies remain fixated on TMT demographic characteristics or heterogeneous structures (Wiersema and Bantel, 1992; Sidhu et al., 2020; Mehrabi et al., 2021), without examining the structural characteristics within teams. Accordingly, there is a relative lack of research on how different types of TMT faultlines affect corporate industrial diversification. This paper examines the impact of faultlines, an internal structural feature of a TMT, on corporate industrial diversification. This study deepens the existing literature on the impact of top management teams on corporate industrial diversification (Guerras-Martin et al., 2020; Ma S. H. et al., 2022). Secondly, integrating faultline theory and the attention-based view, this paper provides a micro-level explanation mechanism for corporate industrial diversification (Elia et al., 2021). This study introduces the micro-level theoretical construct of strategic attentional breadth and demonstrates its mediating role between TMT faultlines and corporate industrial diversification. By extending the strategic attention focus (Wu and Zhao, 2012; Ridge et al., 2017) to strategic attentional breadth, this study responds to Eklund and Mannor’s (2021) call and introduces this theoretical logic to the mechanisms which explain the logical relationship between top management internal structural features and corporate industrial diversification. Thus, this research enriches and develops the theoretical understanding of the relationship between TMT and diversification decisions.

## 2. Theoretical background and hypotheses development

### 2.1. Top management team faultlines and corporate industrial diversification

#### 2.1.1. Top management team faultlines

The term “faultlines” comes from the field of geology and refers to areas of stratigraphic fractures that are prone to occur during earthquakes. In this study, the term TMT faultlines refers to “hypothetical dividing lines” (Lau and Murnighan, 1998), which are based on one or more TMT structural features. These faultlines divide TMTs into several differentiated subgroups that are homogeneous in themselves and heterogeneous in each other (Ma H. J. et al., 2022). In this study, the TMT faultlines are divided into two categories, as follows: task-related TMT faultlines and relationship-related TMT faultlines. The above classifications are the most dominant in the current study of TMT faultlines (Richard et al., 2019; Ma et al., 2021; Ma H. J. et al., 2022). In this study, the intensity of task-related TMT faultlines is defined as the strength of the degree to which subgroups are formed, based on one or more task-related attributes (e.g., functional background and tenure). The intensity of relationship-related TMT faultlines is defined as the degree to which subgroups are formed based on one or more physiological characteristic-related attributes (e.g., gender, age, and education level) (Hutzschenreuter and Horstkotte, 2013; Richard et al., 2019).

#### 2.1.2. Task-related TMT faultlines and corporate industrial diversification

Lau and Murnighan (1998) proposed the demographic faultline theory, which suggests that multiple demographic characteristics may simultaneously influence the generation of team subgroups. Considering that informational decision-making and social categorization perspectives are the basis of demographic faultline theory (van Knippenberg et al., 2004; Thatcher and Patel, 2012), and consistent with previous research (Richard et al., 2019; Barroso-Castro et al., 2022), this study applies these perspectives to the analysis of TMT faultlines and corporate industrial diversification.

Based on an informational decision-making perspective, this study argues that, as the intensity of task-related TMT faultlines increases, the degree of corporate industrial diversification will also increase. Firstly, the presence of task-related TMT faultlines allows for fuller communication within subgroups, forming a diverse information and knowledge base. Task-related faultlines, formed by categorizing team members on the basis of their background and tenure, may generate different sub-groups. However, because the members within the sub-groups have similar functional backgrounds, they are likely to share and support each other’s information, perspectives and expertise as a consortium, rather than basing their support on individual identities or cultural caste (Chung et al., 2015; Xie et al., 2022). Stasser et al. (1989) found that information is shared more freely and fully when subgroup members find that at least one other member holds a similar view, especially in subgroups working under large teams. Therefore, subgroup insiders believe that there are natural allies within their small group; the insiders will support the views of their allies, and even expect these allies to help win over other voices in the TMT. As such, they tend to more freely express their views in discussions (Gibson and Vermeulen, 2003; Richard et al., 2019).

In addition, when faultlines are formed based on task attributes, the heterogeneous knowledge and information exchange among different subgroups is better, broadening the information and knowledge of the TMT. Different task subgroups of a TMT are less likely to generate either relationships or relational conflicts between the members (Jehn et al., 1999). Instead, while the team members are grounded in sharing homogeneous knowledge within their own subgroups, they are more likely to share heterogeneous knowledge and information with other subgroups. This enriches the whole team’s knowledge base and facilitates the TMT making diversified decisions with comprehensive creativity (Chung et al., 2015). When TMT members recognize and respect the expertise and contributions of other subgroup members, this will generate a more positive attitude overall toward task-related diversity. Therefore, the TMT is more likely to value and utilize the diverse knowledge and capabilities of all members in the product expansion process (Bezrukova et al., 2009; Hutzschenreuter and Horstkotte, 2013). In addition, the categorization-elaboration model (CEM) also helps to understand how subgroups that are formed based on task attributes can have beneficial effects (van Knippenberg et al., 2004). The CEM also suggests that the exchange of information and efficient discussions among subgroups can improve team innovation and decision quality (Shin and You, 2022). When team members approach the same task from different perspectives, they gather more knowledge and information, and ultimately explore more possible paths and decision options. In order to integrate different perspectives, team members need to re-evaluate their own positions and deeply analyze and understand the arguments of other subgroups (Richard et al., 2019). These actions combine to provide TMT members with a deeper and more thorough understanding of industrial diversification decisions, as well as potential problems, and possible solutions. This clearer understanding, in turn, enables a better response to the increased demand for information processing associated with the complex task of expanding into new industry sectors. As such, TMTs objectively provide companies with varied and innovative expansion paths, thus contributing to the degree of industrial diversification.

In summary, we believe that the task-related TMT faultlines formed around functional backgrounds and tenure not only improve the information richness and knowledge accessibility of TMT; they also promote benign task-based debates among TMT members. Such debates are beneficial to the complicated information processing required for industrial diversification, which in turn will facilitate the implementation of diversification strategies. Therefore, the following hypothesis is proposed:

*H1a*: Ceteris paribus, increased task-related TMT faultlines positively affect corporate industrial diversification.

#### 2.1.3. Relationship-related TMT faultlines and corporate industrial diversification

Based on the social identity perspective and social-categorization perspective, this study concludes that, as the intensity of relationship-related TMT faultlines increases, the degree of corporate industrial diversification will decrease. Firstly, high-intensity relationship-related TMT faultlines cause stereotypes based on demographic characteristics; these stereotypes interfere with knowledge and information sharing within the TMT. Members of subgroups that are formed based on social identity attributes have similar histories and social experiences, but these members have significantly different histories and social experiences from those outside the subgroup (Richard et al., 2019). Relationship-related TMT faultlines increase the significance of the differences between subgroups (Lau and Murnighan, 1998) and simultaneously provoke multiple stereotypes. This can reinforce the biased perspectives of members of one TMT subgroup toward other TMT members and their contributions (Shin and You, 2022). Further, strong faultlines that are based on social identity attributes (i.e., gender, age, or education level) may increase communication between members within identity-based subgroups, due to similarity-attraction. Meanwhile, these same strong faultlines may decrease communication between different identity-based subgroups (van Knippenberg et al., 2004; Ma et al., 2021).

In addition, high-intensity relationship-related TMT faultlines can cause intra-TMT relationship conflicts (Carton and Cummings, 2012; Thatcher and Patel, 2012) and inhibit communication between TMT subgroups. When top management teams have strong relationship-related TMT faultlines, subgroup insiders are more likely to exclude other subgroups. This exclusion causes conflict and creates less effective communication, as well as less effective debate and less sharing of knowledge, information and perspectives across groups (Xie et al., 2022). Conversely, when relationship-related TMT faultlines are relatively weak, team fragmentation based on demographic biological attributes is less likely. These weaker faultlines will not generate intense relationship conflicts, thus reducing the likelihood of negative effects on diversification strategies (Polzer et al., 2006). To summarize, strong relational faultlines may reduce communication between subgroups and thus interfere with knowledge and information sharing within the top management team (Cooper et al., 2014), whereas low relational faultlines are less likely to cause such interference (Hutzschenreuter and Horstkotte, 2013).

As a result, the stronger the relationship-related TMT faultlines are, the more they provoke stereotypes, increase prejudice, and potentially lead to relationship conflicts between different subgroups. All of this damages the information sharing process within a TMT. Thus, when relationship-related TMT faultlines are strong, on average, relational faultlines have a negative impact on TMT’s successful response to the increased need for information processing, as a result of industrial diversification. Therefore, the following hypothesis is proposed:

*H1b*: Ceteris paribus, increased relationship-related TMT faultlines negatively affect corporate industrial diversification.

### 2.2. Top management team faultlines and strategic attentional breadth

#### 2.2.1. Strategic attentional breadth

Based on a corporate attention-based view (Ocasio, 1997, 2011), how the attention of a TMT is allocated and regulated is closely related to the formulation of corporate strategy. “Strategic attentional breadth” refers to the number of strategic issues that executives focus upon, and the relative extent to which executives pay attention to each issue (Eklund and Mannor, 2021). It is important to note that, in this study, strategic attention is not referred to at the individual executive level, but at the organizational level, i.e., the socially-structured attention model of decision makers within the organization (Ocasio, 1997). Strategic attention emphasizes that an outcome experienced collectively by the executive team, the distribution of influence and the process of sharing information and making decisions together generate a relatively higher level of attention than in any other area. This is a proven attention orientation (Eklund and Mannor, 2021). A previous study (Li et al., 2013) has shown that relatively broad strategic attention can help managers to look beyond their current environment, allowing them to identify and recognize more opportunities.

#### 2.2.2. Task-related TMT faultlines and strategic attentional breadth

Firstly, the members of subgroups formed based on task-related faultlines have convergent attention within them. They are likely to have shared the same or similar experiences, adhere to a common value code, and are thus more likely to develop a focus on a particular topic. Task-related information is less likely to be ignored if that information is captured by a member of the TMT’s subgroup. Team attention is focused on the selected problem, and the more that members are likely to share and support an idea, the more that idea’s value is demonstrated (Hinsz et al., 1997).

Secondly, the subgroups formed based on task-related faultlines can fully integrate and make use of differentiated information to broaden the strategic attentional breadth of the corporation. Different attentional tendencies among subgroups, together with respect for information, information sharing, and information cross-fertilization among different subgroups, make it more likely that teams will value and utilize the different knowledge and abilities of all members in the product expansion process (Bezrukova et al., 2009; Eklund and Mannor, 2021). In addition, when team members approach a task from different directions, more information is gathered, and more diverse options are eventually explored. In order to integrate different perspectives, team members need to re-assess their positions, understand opposing arguments, and develop a deeper understanding of expansion decisions, possible problems, and alternative solutions (Ridge et al., 2017). Consistent with the valuable role that can be played in decision making by debates around tasks and constructive critical challenges, task-related conflict has been shown to be positively associated with TMT decision quality and cognitive task performance (Certo et al., 2006). As a result, strong task-related faultlines reflect marked differences in task-related knowledge and perspectives between subgroups. These faultlines may encourage intra-team discussions, which increase the strategic attentional breadth of the TMT. From this, the following hypothesis is derived:

*H2a*: Ceteris paribus, increased task-related TMT faultlines positively affect strategic attentional breadth.

#### 2.2.3. Relationship-related TMT faultlines and strategic attentional breadth

Firstly, although information exchange within the subgroups is formed based on relational faultlines, it is difficult to have a deeper insight into a particular issue because of the differences in the relevant characteristics of individuals’ functional backgrounds. Relative to other subgroups, individuals in the same subgroup tend to rate each other higher, while members outside the subgroup tend to be undervalued (Messick and Mackie, 1989). This forms sharp boundaries between subgroups, making it difficult for information to flow smoothly between subgroups (Richard et al., 2019). Members within subgroups can strongly identify with each other, but forming a common focus on a particular issue is difficult, due to differences in information perception and experiential background. In addition, even though subgroups identify with each other, it can be difficult for subgroups to explore the deeper meaning of an issue.

Secondly, high-intensity relational faultlines can lead to relational conflicts between subgroups, causing normal information processing to become disruptive and rendering the task unworkable. High-intensity relational faultlines can lead to the development and escalation of one subgroup’s mistrust of and hostility toward members of other subgroups (Li and Hambrick, 2005); relationship conflict may even occur (Amason, 1996). When this situation develops, executive members turn their attention away from task-related issues (Jehn et al., 1999), thereby making the processing of complex information particularly difficult (Simons and Peterson, 2000). In such cases, a TMT’s processing of new or particularly complex information may be hindered by the increased stress and anxiety associated with relational conflict (Simons and Peterson, 2000). High-intensity relational faultlines imply the formation of subgroups within the same team that are not related to the work. In addition, because subgroups share the same stereotypes and biases, managers’ attention is diverted away from normal work programs and issues to other issues that are not related to the task at hand (Hutzschenreuter and Horstkotte, 2013). In addition, subgroups that are formed by high-intensity relational faultlines are subject to the extremes of both “groupthink” and “team fragmentation.” The formation of subgroups with high heterogeneity between groups and high homogeneity within groups can lead to disruptions and interruptions in normal information processing. This is directly due to intergroup divisions, biases and conflicts, which are reinforced by the high level of homogeneity within groups. This phenomenon causes individuals in the same subgroup to tend to rate each other higher (relative to other subgroups), while members outside the subgroup tend to be underrated (Messick and Mackie, 1989). Thus, sharp boundaries are created between subgroups, making it difficult for information to flow smoothly between subgroups.

In summary, executives with similar characteristics will be attracted to each other, forming faultlines and subgroups based on similar demographic characteristics. This will repeatedly reinforce common thought patterns and effectively ensure that subgroup members focus on the same or similar things. When confronted with different opinions, the members of different subgroups are more likely to develop a tit-for-tat approach to dealing with each other (Ridge et al., 2017). At the same time, in order to achieve the interests of the group, the subgroups often argue for different aspects and solutions of the same issue. This creates a situation of “arguing for the sake of argument,” with members opposing the views of any other group members. In such cases, the goal of attention is often concentrated rather than decentralized, and the strategic attentional breadth is relatively narrow. Therefore, the following hypothesis is proposed:

*H2b*: Ceteris paribus, increased relationship-related TMT faultlines negatively affect strategic attentional breadth.

### 2.3. The mediating role of strategic attentional breadth

Studies have shown that TMT characteristics influence executive perceptions (Hambrick and Mason, 1984), which further influence executives’ strategic decisions (Cho and Hambrick, 2006). Also, TMT perceptions are an intermediate mechanism for the role TMT characteristics play in firms’ strategic decisions and behavior (Buyl et al., 2011). Strategic attention, as a mediating variable between TMT characteristics and strategic decisions, can unlock the “black box” between TMT characteristics and corporate industrial diversification decisions. Strategic attention can also uncover the underlying mechanisms (Cho and Hambrick, 2006). On this basis, this study further suggests that strategic attentional breadth acts as an intermediate mechanism between the strength of TMT faultlines and corporate industrial diversification.

Based on the attention-based view, the way in which strategic attentional breadth affects industrial diversification can be viewed as a three-step information processing procedure: “attention, interpretation, and decision making” (Ocasio, 1997; Ridge et al., 2017). Firstly, the strategic attentional breadth of a TMT’s configuration, in terms of the range of issues and answers in the external environment, represents the breadth and depth of that TMT’s ability to focus on emerging and potential market needs, relevant information and expansion opportunities. Secondly, based on these observed market demands, information and opportunities, sense-making and sense-giving are conducted. In other words, on the one hand, the impact of demands, information and opportunities on diversification decisions is constructed and explained in relation to the company’s internal strategic resources and actual situation. On the other hand, potential strategic implementation plans are considered, and the potential problems and solutions of various strategic implementation paths are explained. Thirdly, based on the interpretation and evaluation of strategy implementation options, the TMT’s diversification strategy decisions are influenced.

As TMTs allocate a wider range of issues and answers to the elements of the external environment, they are able to focus more on market needs, information, resources, and expansion opportunities. They can especially focus on the needs of key stakeholders and the important strategic resources that the firm urgently needs (Eklund and Mannor, 2021). In addition, the TMT will use strategic thinking to exploit market opportunities, resource elements and government policies. The TMT will also avoid threats, in order to enhance the firm’s competitive advantage (Li et al., 2013; Ocasio and Joseph, 2018), thereby stimulating the TMT’s intention to diversify and expand. Previous studies have also shown that, when the strategic attentional breadth of a TMT is narrower, this indicates that the TMT’s knowledge is narrow and highly concentrated. The information sources are also more refined but not broad enough. Conversely, when TMT’s strategic attentional breadth is wider, it indicates that they have more diverse information sources and are able to observe more strategic resources that the business needs. This situation also enables a TMT to develop more diverse and creative strategic solutions, and to uncover more potential problems and solutions. Therefore, TMT faultlines will affect the degree of industrial diversification by influencing strategic attentional breadth.

Combining the above analysis, on the basis of Hypothesis 1 and Hypothesis 2, this paper further proposes the hypotheses that:

*H3a*: Ceteris paribus, strategic attentional breadth mediates the relationship between task-related TMT faultlines and corporate industrial diversification.

*H3b*: Ceteris paribus, strategic attentional breadth mediates the relationship between relationship-related TMT faultlines and corporate industrial diversification.

**Figure 1:**
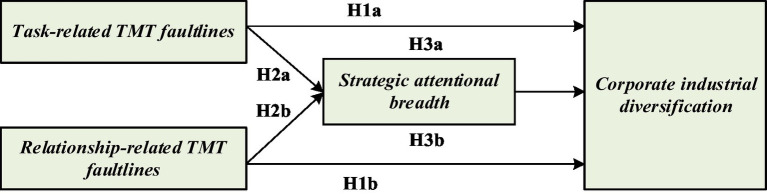
Conceptual framework.

The conceptual framework of this paper is shown in Figure 1.

## 3. Research design

### 3.1. Sample and data

This study uses Chinese A-share listed firms, with data from 2008 to 2021, as the study sample. Since 2008, the China Securities Regulatory Commission (CSRC) has required listed firms to disclose detailed information on TMT members’ demographic characteristics in the firms’ annual reports. Therefore, 2008 is set as the starting year of the sample in this study. Due to the specificity of the financial statements, the sample of firms from the financial industry (CSRC industry code: J) have been excluded (Zhang et al., 2021). Considering that the implementation of industrial diversification strategies usually has a certain lag, and to avoid the endogeneity problem of reverse causality, this study sets a two-period time lag for the regression model. Finally, after excluding samples with missing data pertaining to variables, this study obtained an unbalanced panel sample of 10,972, involving data from 2,063 non-financial listed firms during the years from 2008 to 2019. The distribution of the industries to which the sample firms belong is shown in Appendix Table S1.

This study obtained research data from multiple sources. First, the primary data for calculating corporate industrial diversification are from the Wind Financial Terminal (WFT). Second, the textual data of management discussion and analysis (MD&A) used to calculate strategic attentional breadth were obtained from the listed firms’ annual reports. Third, the demographic characteristics data used to calculate TMT faultlines are from the China Stock Market & Accounting Research (CSMAR) database. Fourth, the data pertaining to firm basic information, financial indicators, and board members have also been obtained from the Wind and CSMAR databases. Finally, to prevent any extreme data from interfering with the analysis, the continuous variables were Winsorized at the upper and lower 1% levels (Chen et al., 2022b).

### 3.2. Measurement

#### 3.2.1. Dependent variable

The dependent variable in this study is *industrial diversification*. Most previous studies use a dummy variable approach to measure industrial diversification, separating firms that report revenues from only one industry from those that report revenues from multiple industries (Rodriguez-Perez and van Hemmen, 2010; Berrill et al., 2021). Referring to previous studies (Li et al., 2012; Sun et al., 2017; Jafarinejad et al., 2018; Schommer et al., 2019; Berrill et al., 2021), this study measures industrial diversification using the Herfindahl measure, which captures the degree of industrial diversification within the firm taking into account the relative importance of industrial segments, thus providing a more accurate ranking of firms than the dummy variable approach. Specifically, the sales revenue of each business of the firm is combined according to the CSRC’s three-digit industry codes. Then, the proportion of the firm’s sales revenue in each industry to the total sales revenue is calculated. Finally, corporate industrial diversification is calculated, according to the following formula:


(1) $$Industrial\;diversification=1-\overset{n}{\underset{i=1}{\sum }}p_{i}^{2}$$

Here, *p_i_* is the proportion of the firm’s sales revenue in industry *i* to total sales revenue; *n* is the number of industries involved in the firm’s business. The larger the value is, the higher is the degree of the corporate industrial diversification. In addition, to ensure the reliability of the research findings, in the robustness test section, this study will also use the entropy measure to recalculate the corporate industrial diversification.

#### 3.2.2. Independent variables

The independent variable in this study is TMT faultlines. Consistent with previous research and theory (Bezrukova et al., 2009; Carton and Cummings, 2012; Chung et al., 2015; Richard et al., 2019), this study classifies TMT faultlines into *task-related TMT faultlines* and *relationship-related TMT faultlines*. Among them, the indicators for constructing task-related TMT faultlines include the functional background and tenure of TMTs. Indicators for constructing relationship-related TMT faultlines include the gender, age, and education level of TMT members. Although many studies categorize education level as a task-related attribute (Hutzschenreuter and Horstkotte, 2013), education level is also one of the most important determinants of subjective social status. Education level may also represent socioeconomic status in the Chinese context, as it reflects Confucian values that emphasize education (Richard et al., 2019). The functional background, tenure, gender, age, and education level of the TMT members are measured as follows.

Functional background is divided into three categories: output roles (R&D, design, and marketing), conversion roles (production and management), and support roles (human resources, finance, and legal). Tenure refers to the number of years from the date of the member’s employment with the firm to the date of observation (Chen et al., 2022b). Gender is simply divided into male and female. Age refers to the age of the member as disclosed in the firm’s annual report. Education level is coded as follows: 1 = high school or below; 2 = college; 3 = university; 4 = master’s degree, and 5 = Ph.D (Zhu, 2013).

This study measured both task-related TMT faultlines and relationship-related TMT faultlines using the *Fau_g_* method proposed by Thatcher et al. (2003). This method has been widely used by other researchers when studying team faultlines (Bezrukova et al., 2009; Hutzschenreuter and Horstkotte, 2013; Zhang and Ma, 2022). Since larger-scale TMTs involve an exponential multiplication of computation, Thatcher et al. (2003) suggested dividing the team into two subgroups. Before further calculations, this study separately pre-processed the nominal and continuous variables (Thatcher et al., 2003). Here, tenure, age and education level were recoded based on the maximum difference normalization method. Functional background and gender were processed by rescaling, i.e., generating a corresponding number of dummy variables based on categories, then dividing by $\sqrt{2}$. Ultimately, TMT faultlines were calculated using the following formula:


(2) $$Fau_{g}=\frac{\sum_{j=1}^{q}\sum_{k=1}^{2}n_{k}^{g}{\left({\bar{x}}_{jk}-{\bar{x}}_{j}\right)}^{2}}{\sum_{j=1}^{q}\sum_{k=1}^{2}\sum_{i=1}^{n_{k}^{g}}{\left(x_{ijk}-{\bar{x}}_{j}\right)}^{2}}$$

Here, *q* represents the number of demographic characteristics involved in a given TMT faultlines, and *g* represents the number of group divisions. For a group of size *n*, there are 2^*n*-1^ classifications divided into two subgroups. Then, ${\bar{x}}_{j}$ represents the average of the characteristics *j* of the TMT members; ${\bar{x}}_{jk}$ represents the average of the characteristics *j* of the TMT members in the *k*-th subgroup; $x_{ijk}$ represents the value of the *i*-th member of the *k*-th subgroup on characteristic *j*, and $n_{k}^{g}$ represents the number of TMT members in the *k*-th subgroup of the TMT team in the *g*-th classification. After going through the *Fau_g_* values under all classification methods (2^*n*-1^ in total), the largest *Fau_g_* value was selected as the measure of TMT faultlines in this study. The calculation of the TMT faultlines was conducted by writing the corresponding program in Matlab R2021a software. For the definition of TMT members, consistent with the definition in the CSMAR database, this study defines a TMT member as one who serves as senior management in the firm, as disclosed in the firm’s annual report, excluding members of the board of directors and supervisory board.

#### 3.2.3. Mediating variable

The mediating variable in this study is *strategic attentional breadth*. In this study, the textual analysis method is used to code the textual content of the MD&A section of firms’ annual reports, to measure the firm’s attention to each strategy category. Then, the Herfindahl measure is used to calculate the strategic attentional breadth (Eklund and Mannor, 2021). Specifically, the calculation process includes the following three steps:

Step 1: Conduct word frequency analysis on the text content of the MD&A. Drawing on Eklund and Mannor (2021), this study uses the dictionary approach to objectively capture firms’ allocation of attention to 13 strategy categories, such as alliance partner strategies (APS). Based on the textual content of the MD&A section of listed firms’ annual reports, this study modified the dictionary words, based on Eklund and Mannor (2021), to conform to the Chinese expressions. Only meaningful words with a certain occurrence frequency were retained (see Appendix Table S2 for example dictionary words).

Step 2: Calculate the allocation of attention to the 13 strategy categories in the MD&A. This study calculated the firm’s attention to strategy category *i* as the cumulative value of the product of each word’s length and its occurrence frequency.

Step 3: Calculate strategic attentional breadth using the Herfindahl measure. This study calculated the proportion of the firm’s attention in each strategy category to the attention of all strategy categories. Finally, strategic attentional breadth is calculated using the following formula:


(3) $$Strategic\;attention\;breadth=1-\overset{n}{\underset{i=1}{\sum }}a_{i}^{2}$$

Here, *a_i_* is the proportion of the firm’s attention in strategy category *i* to the attention of all strategy categories, and *n* is the number of strategic categories. The higher the value is, the greater is the strategic attentional breadth. In addition, to ensure the reliability of the research findings, in the robustness test section, this study will use the entropy measure to recalculate the strategic attentional breadth.

#### 3.2.4. Control variables

To more accurately capture the impact of TMT faultlines on corporate industrial diversification, this study uses controls for the following variables: first, basic characteristics, such as the size and age of the firm, are introduced as control variables. Firm size is often seen as an indicator of scale economies, and a strong link is known to exist between firm size and the level of diversification (Grant et al., 1988; Wang and Luo, 2019). Older firms are less inclined to pursue industrial diversification, because they are less flexible and more closely tied to existing businesses (Coad and Guenther, 2013). The variable *firm size* is taken as the number of employees (*ln*) of the firm, while *firm age* is measured according to the length of time between the year the firm was established and the observation year.

Second, this study controls for a firm’s financial resources, including leverage ratio and current ratio (Hutzschenreuter and Horstkotte, 2013; Chen et al., 2023). Previous research has shown that industrial diversification can be financed by increasing leverage (Kochhar and Hitt, 1998). *Leverage ratio* is measured using the ratio of assets to liabilities. *Current ratio* is measured using the ratio of current assets to liabilities (Chen et al., 2023).

Third, this study controls for the relationship between a firm and the state or government, i.e., the nature of ownership. To this end, *state ownership* was set to 1 if the state owned or controlled the firm’s capital; 0 otherwise (Zhou et al., 2017).

Forth, this study controls for board independence, because it is a strong proxy for corporate governance and is thought to influence corporate industrial diversification (Sun et al., 2017). *Board independence* is a proportional variable that is based on the ratio of the number of independent directors to the total number of members of the firm’s board of directors.

Finally, the potential impact of *industrial competition* is also considered. Like Xu et al. (2019) and Chen et al. (2022a), this study measures industry competition by using the following formula: 1 minus a Herfindahl measure, based on the sales revenue of each listed firm within the CSRC three-digit industry.

### 3.3. Modeling

This study used unbalanced panel data to test the hypotheses. First, a Hausman test was used to determine which model is more appropriate, the fixed-effects or random-effects model. The results of the Hausman test show the chi-square value of 227.29, corresponding to the value of p of less than 0.01. Therefore, this study used the fixed-effects model for hypothesis testing (Chen et al., 2022a). To test Hypotheses 1a and 1b, this study established the following fixed-effects regression model:


(4) $$\begin{array}{c} Industrial\;diversification_{i,t+2}=\beta_{0}+\beta_{1}TMT\;faultlines_{i,t}+\beta_{2}X_{i,t}\\ \;\;\;\;\;\;\;\;\;\;\;\;\;\;\;\;\;\;\;\;\;\;\;\;\;\;\;\;\;\;\;+\mu +\lambda +\gamma +\varepsilon_{i,t} \end{array}$$

Here, *industrial diversification* is the dependent variable; *TMT faultlines* are the independent variables, including task-related TMT faultlines and relationship-related TMT faultlines; *X* represents a set of control variables; *μ*, *γ* and *λ* represent firm, year, and industry dummy variables, respectively, and *ε* is the residual term.

To test Hypotheses 2a and 2b, this study established the following fixed-effects regression model:


(5) $$\begin{array}{c} Strategic\;attentional\;breadth_{i,t+1}=\beta_{0}+\beta_{1}TMT\;faultlines_{i,t}+\beta_{2}X_{i,t}\\ \;\;\;\;\;\;\;\;\;\;\;\;\;\;\;\;\;\;\;\;\;\;\;\;\;\;\;\;\;\;\;\;\;\;\;\;\;\;\;+\mu +\lambda +\gamma +\varepsilon_{i,t} \end{array}$$

Here, *strategic attentional breadth* is the dependent variable. Following the practice of previous study (Cho and Hambrick, 2006), this study sets the observation period of strategic attentional breadth between TMT structural characteristics and firm strategic. Specifically, this study sets the observation period of strategic attentional breadth at period *t* + 1, making it later than the observation period of TMT faultlines (period *t*) and before the observation period of industrial diversification (period *t* + 2), thus helping to mitigate reverse causality. The other variables are set in line with Equation 4 and are not repeated here.

To test Hypotheses 3a and 3b, this study established the following fixed-effects regression model:


(6) $$\begin{array}{c} Industrial\\ diversification_{i,t+2} \end{array}=\begin{array}{c} \beta_{0}+\beta_{1}TMT\;faultlines_{i,t}\\ \;\;+\beta_{2}Strategic\;attentional\;breadth_{i,t+1}+\beta_{3}X_{i,t}\\ +\mu +\lambda +\gamma +\varepsilon_{i,t} \end{array}$$

Here, *industrial diversification* is the dependent variable. Compared to Equation 4, strategic attentional breadth is introduced here as an additional control variable, to test the mediating role of strategic attentional breadth in the relationship between TMT faultlines and industrial diversification.

## 4. Results

Table 1 presents the descriptive statistics and correlation coefficients of the variables. In this study, the VIF test was performed on all variables entering the model. The results show that the range of VIF values was between 1.01 and 1.92, which did not exceed 10, the threshold of multicollinearity. Therefore, no serious multicollinearity problem exists in this study.

**Table 1:** Descriptive statistics and correlation coefficients.

	Variables	1	2	3	4	5	6	7	8	9	10	11
1	Industrial diversification	1.000										
2	Task-related TMT faultlines	0.012	1.000									
3	Relationship-related TMT faultlines	0.019	0.057	1.000								
4	Strategic attentional breadth	0.038	−0.001	0.023	1.000							
5	Firm size	0.064	−0.071	−0.075	−0.015	1.000						
6	Firm age	0.085	−0.032	0.039	−0.098	0.091	1.000					
7	Leverage ratio	0.083	−0.057	−0.031	−0.052	0.368	0.187	1.000				
8	Current ratio	−0.096	0.038	0.029	0.025	−0.361	−0.171	−0.661	1.000			
9	State ownership	0.096	−0.030	−0.012	−0.065	0.312	0.147	0.319	−0.240	1.000		
10	Board independent	0.008	0.015	0.037	0.008	0.025	−0.032	−0.001	0.033	−0.069	1.000	
11	Industry competition	0.001	0.012	0.007	−0.024	−0.039	0.095	−0.014	0.007	−0.094	−0.018	1.000
	Mean	0.242	0.680	0.799	0.863	7.657	16.064	0.420	2.679	0.346	0.373	0.891
	S.D.	0.245	0.154	0.118	0.023	1.236	5.886	0.211	2.738	0.476	0.054	0.118
	Min	0.000	0.429	0.499	0.738	3.555	2.986	0.054	0.215	0.000	0.000	0.236
	Max	0.829	1.000	0.988	0.898	10.968	32.356	1.201	16.123	1.000	0.571	0.984

*N* = 10,972; the absolute values of all the correlations not less than 0.025 are significant at 0.01.

### 4.1. Test of hypotheses 1a and 1b

This section provides regression results for the relationship between TMT faultlines and corporate industrial diversification. Table 2 focuses on task-related TMT faultlines, while Table 3 focuses on relationship-related TMT faultlines.

**Table 2:** Panel fixed-effects regression of the relationship between task-related TMT faultlines and corporate industrial diversification.

Model number	Model 1	Model 2	Model 3	Model 4
Dependent variable	Industrial diversification	Strategic attentional breadth	Industrial diversification	Industrial diversification
Task-related TMT faultlines	2.593**	0.388***		2.517**
	(2.37)	(2.66)		(2.30)
Strategic attentional breadth			0.201**	0.195**
			(2.51)	(2.44)
Firm size	−0.024	0.130***	−0.054	−0.049
	(−0.07)	(2.82)	(−0.15)	(−0.14)
Firm age	−3.229***	0.099	−3.266***	−3.248***
	(−4.67)	(1.08)	(−4.73)	(−4.70)
Leverage ratio	−0.647	−0.501**	−0.575	−0.549
	(−0.40)	(−2.35)	(−0.36)	(−0.34)
Current ratio	−0.258***	−0.028**	−0.253***	−0.252***
	(−2.84)	(−2.30)	(−2.79)	(−2.78)
State ownership	−1.777	0.344*	−1.789	−1.845
	(−1.34)	(1.96)	(−1.35)	(−1.40)
Board independent	9.481**	0.937*	9.438**	9.298**
	(2.35)	(1.75)	(2.34)	(2.30)
Industry competition	−1.159	0.757**	−1.381	−1.307
	(−0.40)	(1.98)	(−0.48)	(−0.45)
Constant	80.393***	84.641***	65.849***	63.853***
	(6.38)	(50.52)	(4.61)	(4.47)
Firm, year and industry fixed effects	Yes	Yes	Yes	Yes
Adj *R*^2^	0.758	0.491	0.758	0.759
*F*-value	7.669***	30.223***	7.679***	7.651***
Observations	10,972	10,972	10,972	10,972
Firms	2,063	2,063	2,063	2,063

*p < 0.10, **p < 0.05, ***p < 0.01; *t* statistics in parentheses; two-tailed tests; Regression coefficient is expanded 100 times. The Sobel test shows that the Z-statistic of the mediating effect is 1.999 (*p* < 0.05).

**Table 3:** Panel fixed-effects regression of the relationship between relationship-related TMT faultlines and corporate industrial diversification.

Model number	Model 1	Model 2	Model 3	Model 4
Dependent variable	Industrial diversification	Strategic attentional breadth	Industrial diversification	Industrial diversification
Relationship-related TMT faultlines	−3.586**	−0.500**		−3.488**
	(−2.26)	(−2.37)		(−2.20)
Strategic attentional breadth			0.201**	0.196**
			(2.51)	(2.45)
Firm size	−0.064	0.124***	−0.054	−0.088
	(−0.18)	(2.70)	(−0.15)	(−0.25)
Firm age	−3.230***	0.099	−3.266***	−3.249***
	(−4.67)	(1.08)	(−4.73)	(−4.70)
Leverage ratio	−0.595	−0.494**	−0.575	−0.499
	(−0.37)	(−2.31)	(−0.36)	(−0.31)
Current ratio	−0.255***	−0.027**	−0.253***	−0.249***
	(−2.81)	(−2.26)	(−2.79)	(−2.75)
State ownership	−1.755	0.348**	−1.789	−1.823
	(−1.33)	(1.98)	(−1.35)	(−1.38)
Board independent	9.692**	0.968*	9.438**	9.502**
	(2.40)	(1.80)	(2.34)	(2.36)
Industry competition	−1.325	0.733*	−1.381	−1.469
	(−0.46)	(1.92)	(−0.48)	(−0.51)
Constant	85.540***	85.385***	65.849***	68.791***
	(6.79)	(50.93)	(4.61)	(4.80)
Firm, year and industry fixed effects	Yes	Yes	Yes	Yes
Adj R^2^	0.758	0.491	0.758	0.759
F-value	7.663***	30.199***	7.679***	7.645***
Observations	10,972	10,972	10,972	10,972
Firms	2,063	2,063	2,063	2,063

*p < 0.10, **p < 0.05, ***p < 0.01; *t* statistics in parentheses; two-tailed tests; Regression coefficient is expanded 100 times. The Sobel test shows that the Z-statistic of the mediating effect is-1.894 (*p* < 0.10).

Hypothesis 1a of this paper proposes that, *ceteris paribus*, increased task-related TMT faultlines positively affect corporate industrial diversification. According to Model 1 in Table 2, the regression coefficient of task-related TMT faultlines on corporate industrial diversification is significantly positive (*β* = 2.593, *p* < 0.05). The above results show that, for each standard deviation (0.154) increase in task-related TMT faultlines, corporate industrial diversification will increase by 0.016 standard deviations (i.e., 2.593*0.154/0.245/100). Therefore, Hypothesis 1a is supported.

Hypothesis 1b of this paper proposes that, *ceteris paribus*, increased relationship-related TMT faultlines negatively affect corporate industrial diversification. According to Model 1 in Table 3, the regression coefficient of relationship-related TMT faultlines on corporate industrial diversification is significantly negative (*β* = −3.586, *p* < 0.05). The above results show that, for each standard deviation (0.118) increase in relationship-related TMT faultlines, corporate industrial diversification will decrease by 0.017 standard deviations (i.e., −3.586*0.118/0.245/100). Therefore, Hypothesis 1b is supported.

### 4.2. Test of hypotheses 2a and 2b

This section provides regression results for the relationship between TMT faultlines and strategic attentional breadth. Table 2 focuses on task-related TMT faultlines, while Table 3 focuses on relationship-related TMT faultlines.

Hypothesis 2a of this paper proposes that, *ceteris paribus*, increased task-related TMT faultlines positively affect strategic attentional breadth. According to Model 2 in Table 2, the regression coefficient of task-related TMT faultlines on strategic attentional breadth is somewhat significantly positive (*β* = 0.388, *p* < 0.01). The above results show that, for each standard deviation (0.154) increase in task-related TMT faultlines, strategic attentional breadth will increase by 0.026 standard deviations (i.e., 0.388*0.154/0.023/100). Therefore, Hypothesis 2a is partially supported.

Hypothesis 2b of this paper proposes that, *ceteris paribus*, increased relationship-related TMT faultlines negatively affect strategic attentional breadth. According to Model 2 in Table 3, the regression coefficient of relationship-related TMT faultlines on strategic attentional breadth is significantly negative (*β* = −0.500, *p* < 0.05). The above results show that, for each standard deviation (0.118) increase in relationship-related TMT faultlines, strategic attentional breadth will decrease by 0.026 standard deviations (i.e., −0.500*0.118/0.023/100). Therefore, Hypothesis 2b is supported.

### 4.3. Test of hypotheses 3a and 3b

This section provides regression results with regard to the mediating role of strategic attentional breadth in the relationship between TMT faultlines and corporate industrial diversification. Table 2 focuses on task-related TMT faultlines, while Table 3 focuses on relationship-related TMT faultlines. According to Model 3 in Table 2 (or Table 3), the regression coefficient of strategic attentional breadth on corporate industrial diversification is significantly positive (*β* = 0.201, *p* < 0.05). Combining this result with the degree of support for the above hypotheses, there may be an indirect effect of strategic attentional breadth, which will be further tested below.

Hypothesis 3a of this paper proposes that, *ceteris paribus*, strategic attentional breadth mediates the relationship between task-related TMT faultlines and corporate industrial diversification. According to Model 4 in Table 2, after controlling for strategic attentional breadth, the regression coefficient of task-related TMT faultlines on corporate industrial diversification is still significantly positive (*β* = 2.517, *p* < 0.05). However, the regression coefficient decreases compared to Model 1 in Table 2. In addition, the Sobel test shows that the Z-statistic of the mediating effect is 1.999, which is significant at the 5% level. The above results indicate that strategic attentional breadth plays a partially-mediating role in the relationship between task-related TMT faultlines and corporate industrial diversification, with the magnitude of the mediating role being 2.92% (i.e., 0.388*0.195/2.593*100%). Therefore, Hypothesis 3a is supported.

Hypothesis 3b of this paper proposes that, *ceteris paribus*, strategic attentional breadth mediates the relationship between relationship-related TMT faultlines and corporate industrial diversification. According to Model 4 in Table 3, after controlling for strategic attentional breadth, the regression coefficient of relationship-related TMT faultlines on corporate industrial diversification is still significantly negative (*β* = −3.488, *p* < 0.05). However, the absolute value of the regression coefficient decreases, compared to Model 1 in Table 3. In addition, the Sobel test shows that the Z-statistic of the mediating effect is-1.894, which is significant at the 10% level. The above results indicate that strategic attentional breadth plays a partially mediating role in the relationship between relationship-related TMT faultlines and corporate industrial diversification, with the magnitude of the mediating role being 2.73% (i.e., −0.500*0.196/−3.586*100%). Therefore, Hypothesis 3b is supported.

### 4.4. Robustness test

First, the measure of corporate industrial diversification and strategic attentional breadth is replaced. Instead of the Herfindahl measure used in the above section, the entropy measure (Chen et al., 2019; Jiao et al., 2020) is now used to re-measure the corporate industrial diversification and strategic attentional breadth. The respective formulas are shown in Equation 7 and Equation 8.


(7) $$Industrial\;diversification=\overset{n}{\underset{i=1}{\sum }}p_{i}{}^{*}\ln\left(1/p_{i}\right)$$

Here, *p_i_* is the proportion of the firm’s sales revenue in industry *i* to total sales revenue; *n* is the number of industries involved in the firm’s business. The larger the value is, the higher is the degree of corporate industrial diversification.


(8) $$Strategic\;attention\;breadth=\overset{n}{\underset{i=1}{\sum }}a_{i}{}^{*}\ln\left(1/a_{i}\right)$$

Here, *a_i_* is the proportion of the firm’s attention in strategy category *i* to the attention of all strategy categories, and *n* is the number of strategic categories. The higher the value is, the greater is the strategic attentional breadth.

After changing the measurement method, the obtained findings remain unchanged; the regression results are presented in Tables 4, 5.

**Table 4:** Panel fixed-effects regression of the relationship between task-related TMT faultlines and corporate industrial diversification (measuring industrial diversification and strategic attentional breadth using the Entropy Measure).

Model number	Model 1	Model 2	Model 3	Model 4
Dependent variable	Industrial diversification	Strategic attentional breadth	Industrial diversification	Industrial diversification
Task-related TMT faultlines	3.919**	1.764***		3.710**
	(2.15)	(2.80)		(2.04)
Strategic attentional breadth			0.120***	0.119***
			(3.92)	(3.86)
Control variables	Yes	Yes	Yes	Yes
Constant	119.797***	209.633***	98.130***	94.933***
	(5.73)	(29.00)	(4.50)	(4.34)
Firm, year and industry fixed effects	Yes	Yes	Yes	Yes
Adj *R*^2^	0.797	0.532	0.798	0.798
*F*-value	9.538***	38.203***	9.694***	9.624***
Observations	10,972	10,972	10,972	10,972
Firms	2,063	2,063	2,063	2,063

*p < 0.10, **p < 0.05, ***p < 0.01; *t* statistics in parentheses; two-tailed tests; Regression coefficient is expanded 100 times. The Sobel test shows that the Z-statistic of the mediating effect is 2.518 (*p* < 0.05).

**Table 5:** Panel fixed-effects regression of the relationship between relationship-related TMT faultlines and corporate industrial diversification (measuring industrial diversification and strategic attentional breadth using the Entropy Measure).

Model number	Model 1	Model 2	Model 3	Model 4
Dependent variable	Industrial diversification	Strategic attentional breadth	Industrial diversification	Industrial diversification
Relationship-related TMT faultlines	−4.418*	−1.944**		−4.186
	(−1.68)	(−2.14)		(−1.59)
Strategic attentional breadth			0.120***	0.119***
			(3.92)	(3.88)
Control variables	Yes	Yes	Yes	Yes
Constant	126.839***	212.769***	98.130***	101.442***
	(6.06)	(29.40)	(4.50)	(4.63)
Firm, year and industry fixed effects	Yes	Yes	Yes	Yes
Adj *R*^2^	0.797	0.532	0.798	0.798
*F*-value	9.511***	38.145***	9.694***	9.601***
Observations	10,972	10,972	10,972	10,972
Firms	2,063	2,063	2,063	2,063

*p < 0.10, **p < 0.05, ***p < 0.01; *t* statistics in parentheses; two-tailed tests; Regression coefficient is expanded 100 times. The Sobel test shows that the Z-statistic of the mediating effect is-2.079 (*p* < 0.05).

Second, the Heckman model is used to deal with the problem of sample selection bias, due to missing data on TMT demographic characteristics. In this study, approximately 49.1% of the observations did not contain reports of TMT demographic characteristics that would be sufficient to measure TMT faultlines. This could potentially lead to sample selection bias (Heckman, 1979). To exclude the adverse effects of missing TMT faultlines data, this study used the Heckman two-stage model to correct for missing TMT faultlines; the regression results are shown in Tables 6, 7.

**Table 6:** Panel fixed-effects regression of the relationship between task-related TMT faultlines and corporate industrial diversification (using the Heckman model to mitigate sample selection bias).

Model number	Model 1	Model 2	Model 3	Model 4	Model 5
Dependent variable	Sufficient TMT information	Industrial diversification	Strategic attentional breadth	Industrial diversification	Industrial diversification
Regression stage	First stage	Second stage	Second stage	Second stage	Second stage
Industry average of sufficient TMT information	2.667***				
	(18.44)				
Province average of sufficient TMT information	2.449***				
	(22.76)				
Task-related TMT faultlines		2.592**	0.388***		2.516**
		(2.36)	(2.66)		(2.29)
Strategic attentional breadth				0.201**	0.196**
				(2.51)	(2.45)
Control variables	Yes	Yes	Yes	Yes	Yes
Inverse Mills ratio		−0.272	0.157	−0.281	−0.303
		(−0.22)	(0.97)	(−0.23)	(−0.25)
Constant	−2.161***	80.558***	84.554***	65.999***	64.015***
	(−9.90)	(6.39)	(50.39)	(4.62)	(4.47)
Firm, year and industry fixed effects	—	Yes	Yes	Yes	Yes
Pseudo or Adj *R*^2^	0.105	0.758	0.491	0.758	0.759
Chi2 or *F*-value	3135.678***	7.655***	30.231***	7.666***	7.637***
Observations	21,583	10,969	10,969	10,969	10,969
Firms	2,795	2,062	2,062	2,062	2,062

*p < 0.10, **p < 0.05, ***p < 0.01; *t* statistics in parentheses; two-tailed tests; Regression coefficient is expanded 100 times. The Sobel test shows that the Z-statistic of the mediating effect is 1.774 (*p* < 0.10).

**Table 7:** Panel fixed-effects regression of the relationship between relationship-related TMT faultlines and corporate industrial diversification (using the Heckman model to mitigate sample selection bias).

Model number	Model 1	Model 2	Model 3	Model 4	Model 5
Dependent variable	Sufficient TMT information	Industrial diversification	Strategic attentional breadth	Industrial diversification	Industrial diversification
Regression stage	First stage	Second stage	Second stage	Second stage	Second stage
Industry average of sufficient TMT information	2.667***				
	(18.44)				
Province average of sufficient TMT information	2.449***				
	(22.76)				
Relationship-related TMT faultlines		−3.583**	−0.503**		−3.485**
		(−2.26)	(−2.38)		(−2.20)
Strategic attentional breadth				0.201	0.196**
				(2.51)	(2.46)
Control variables	Yes	Yes	Yes	Yes	Yes
Inverse mills ratio		−0.217	0.165	−0.281	−0.249
		(−0.18)	(1.02)	(−0.23)	(−0.21)
Constant	−2.161***	85.672***	85.295***	65.999***	68.923***
	(−9.90)	(6.79)	(50.79)	(4.62)	(4.80)
Firm, year and industry fixed effects	—	Yes	Yes	Yes	Yes
Pseudo or Adj *R*^2^	0.105	0.758	0.491	0.758	0.759
Chi2 or *F*-value	3135.678***	7.649***	30.208***	7.666***	7.631***
Observations	21,583	10,969	10,969	10,969	10,969
Firms	2,795	2,062	2,062	2,062	2,062

*p < 0.10, **p < 0.05, ***p < 0.01; *t* statistics in parentheses; two-tailed tests; Regression coefficient is expanded 100 times. The Sobel test shows that the Z-statistic of the mediating effect is-1.715 (*p* < 0.10).

Model 1 in Table 6 (or Table 7) shows the first stage of the Heckman regression model, where the dependent variable is *sufficient TMT information*. Specifically, sufficient TMT information takes the value of 1 if the firm reports a sufficient amount of TMT demographic characteristics to measure TMT faultlines; 0 otherwise. As suggested by Wolfolds and Siegel (2019), exclusion restriction variables were introduced in the first stage to improve the validity of the regression analysis. The first exclusion restriction variable is the proportion of peer firm reporting sufficient TMT demographic characteristics to measure TMT faultlines, i.e., *industry average of sufficient TMT information*. The second exclusion restriction variable is the proportion of same-province firms reporting sufficient TMT demographic characteristics to measure TMT faultlines, i.e., *province average of sufficient TMT information*. The results of Model 1 show that both exclusion restriction variables are significantly positive (*p* < 0.01), indicating the validity of the exclusion restriction variable (Chen et al., 2022b).

Finally, the *inverse Mills ratio* was calculated, using the regression results from the first stage. This ratio was then introduced into the second stage for control. As can be seen from the regression results in Tables 6, 7, the findings of the study did not change after using the Heckman model to mitigate the sample selection bias.

## 5. Conclusion and discussion

Based on faultline theory and attention-based view, this paper analyzes the impact of TMT faultlines (task-related TMT faultlines and relationship-related TMT faultlines) on corporate industrial diversification. The research also reveals the channel effect of strategic attentional breadth. After analyzing 10,972 observations of 2,063 non-financial listed companies for the period 2008–2021, the following conclusions were obtained: firstly, task-related TMT faultlines and relationship-related TMT faultlines have completely opposite effects on corporate industrial diversification. Specfically, the former promotes industrial diversification, and the latter inhibits it. Secondly, task-related TMT faultlines and relationship-related TMT faultlines also have an impact on a firm’s strategic attentional breadth. Specifically, the former has a facilitating effect on strategic attentional breadth, while the latter has an inhibiting effect. Third, strategic attentional breadth plays a partially-mediating role in the relationship between both task-related and relationship-related TMT faultlines and corporate industrial diversification.

### 5.1. Theoretical contributions

The contribution of this paper is mainly in two aspects. Firstly, based on faultline theory, this paper provides a new explanation for corporate industrial diversification from the perspective of the internal structural characteristics of TMTs. Early industrial diversification literature mainly used theoretical perspectives beyond the boundaries of the firm to explain the diversification behavior of firms, such as the S-C-P framework and dynamic competition (Guerras-Martin et al., 2020). In recent years, academics have gradually turned to theoretical perspectives within the boundaries of the firm, and in greater depth, from intra-firm resources to agency costs, to top managers’ representations and on to top managers’ perceptions (Neely et al., 2020; Elia et al., 2021). However, there is still a relative lack of research on how different types of TMT faultlines affect corporate industrial diversification. To address this gap in research, unlike existing studies that adopt a single theoretical perspective to explain diversification behavior, this paper integrates faultline theory and the attention-based view in an attempt to provide a step-by-step perspective of executive managers’ diversification behavior. Analyzes are made from the surface to the inside (e.g., from TMT faultlines to strategic attentional breadth). However, the studies that have been conducted specifically at the TMT level have ranged from focusing on the teams’ specific demographic characteristics (e.g., members’ age and tenure; Wiersema and Bantel, 1992; Boeker, 1997) to comprehensive demographic characteristics (e.g., degree of team diversity or heterogeneity; Mehrabi et al., 2021). While these studies are increasingly rich in the information they provide, they still remain focused on external surface characteristics, without inspecting the teams’ internal structural characteristics. In reality, the teams’ internal structural characteristics affect information sharing, group interaction, and competitive games among members (Carton and Cummings, 2012). This makes it possible to more strongly characterize and predict the impact of the phenomenon of “harmony in diversity, sameness but disharmony” on group decision making. In light of the above, this paper deepens the existing literature on the impact of TMT on diversification by examining the impact of faultlines, an internal structural feature of TMT, on corporate industrial diversification.

Secondly, integrating faultline theory and the attention-based view, this paper provides a micro-level explanatory mechanism for corporate industrial diversification. By examining the mediating role of strategic attentional breadth, this paper enriches current literature’s focus on the direct effects of top management on diversification, in terms of research content. A great deal of research has been conducted to explore the direct effects of top management on diversification. However, there is considerably less empirical research on the mechanisms of action (e.g., cognition and attention), by which top management teams influence corporate diversification. Although a number of case studies have tried (and called for) revealing the intermediate mechanisms by which managerial characteristics influence diversity (Cho and Hambrick, 2006), this limitation is still frequently mentioned, even today. Thus, this issue has evolved into an important research direction for the future (Neely et al., 2020). Benefiting from the attention-based view (Ridge et al., 2017; Ocasio and Joseph, 2018), this paper examines the mediating role played by strategic attentional breadth, based on the direct impact of TMT faultlines on corporate industrial diversity. In fact, Wu and Zhao (2012) explained corporate industrial diversification from the perspective of attention allocation. However, unlike their study that concentrated on the focus of strategic attention (allocated to customers, competitors, or employees), this paper concentrates on the breadth of strategic attention. This approach is an extension and expansion from the “point” to the “surface” (Eklund and Mannor, 2021). Besides, the objects of attention in the concept of strategic attentional breadth in this paper are different corporate strategies (e.g., product marketing strategies, resource and capability development strategies, social strategies, etc.). This differs from the objects of attention in the paper by Wu and Zhao (2012), who were people (e.g., customers, competitors or employees). The above two differences or extensions lead to a smoother logical relationship between strategic attentional breadth and the internal structural characteristics of TMT and corporate industrial diversification.

### 5.2. Practical implications

The practical implications of this paper are manifested in the following aspects: firstly, based on the findings of this paper, the degree of corporate industrial diversification can be influenced through the rational allocation of faultlines. The larger the relationship-related TMT faultlines are, the more difficult it is for the TMT to make collective decisions, and the less likely the TMT is to reach agreement on strategic decisions for diversified businesses. In addition, the larger the task-related TMT faultlines are, the more likely it is that “fragmentation” will form within the TMT and extend to the business areas in which the company operates. Therefore, the more cordial the TMT relationship (smaller relationship-related faultlines) is, and the more diverse the executive functions and professional backgrounds (larger task-related faultlines) are, the greater the likelihood that Chinese companies will choose a highly-diversified strategy. Therefore, it is important to pay close attention to the impact of TMT faultlines on corporate industrial diversification. Adjusting TMT composition, positions, and division of labor can help encourage companies to choose moderately-focused diversification strategies. Secondly, this paper shows that the impact effect of TMT faultlines can be achieved through strategic attentional breadth. The external environment can be dynamic, complex and ambiguous. In addition, changes in the external environment may be outside the core knowledge structure of senior management. Obviously, then, making individual decisions in such an environment will be extremely difficult for the CEO or chairman. Thus, it is necessary for a TMT to take advantage of the team’s strengths to “focus” on the core vision, while maintaining the “residual light” in the peripheral vision (Li, 2021). Therefore, companies should give full play to the positive impact of TMT faultlines, in such a way that TMT attention should have both “deep focus” and “broad strategic vision.” For example, around the company’s own core strengths, the TMT can engage in brainstorming from time to time, to explore the possibility of extending and developing its core strengths into other related fields.

### 5.3. Limitations and suggestions for future research

This paper still has the following limitations: first, this study focuses only on industrial diversification, i.e., the number of new industry sectors entered and their relevance to existing industries. Future studies may explore the impact of TMT faultlines on geographic diversification or international expansion. Second, this study focuses on the relationship between the influence of internal TMT structure on strategic attentional breadth and industrial diversification. However, the relationship between faultlines and team performance or firm performance is not analyzed. Studies have shown that, in some cases, task faultlines can indeed serve as “healthy dividing lines” (Gibson and Vermeulen, 2003). However, task faultlines can also trigger task conflict at high levels when they affect the team’s information processing; may positively affect task performance; further research could also consider outcome variables of firm performance to enrich the existing findings. Third, although we analyzed the mediating role of strategic attentional breadth in TMT faultlines and industrial diversification, there is still a large part of unexplained “black box,” which implies room for other theoretical explanations. TMT faultlines may influence industrial diversification through another process other than strategic attentional breadth. Future research could introduce new theories (e.g., strategic decision process theory; Rajagopalan et al., 1993; Elbanna, 2006) that provide additional explanations for the relationship between TMT structure and strategic decisions (e.g., characteristics of the decision process). Fourth, future research can further consider the impact of characteristics of different stages of faultlines formation on firms’ strategic behaviors. In the early stage of subgroup formation, members tend to use some significant biological characteristics, such as gender and age, as the basis for group segmentation (Maznevski, 1994). However, the significance of group demographic faultlines decreases in line with the accumulation of common work experiences and mutual understanding among group members. Instead, the effects of individual beliefs, values, and preferences become more pronounced as a result of interpersonal interactions. Stereotypes, prejudice, and discrimination based on differences in demographic characteristics within the group will gradually decrease, and work-related characteristics may become a strong basis for subgroup segmentation (Harrison et al., 1998). Future research may explore the role of temporal factors (Meister et al., 2020) in the relationship between TMT faultlines and strategic decision-making; the common belief is that very interesting findings will be generated.

## Data availability statement

The raw data supporting the conclusions of this article will be made available by the authors, without undue reservation.

## Author contributions

WC performed conceptualization, methodology, visualization, and writing-original draft. CC did conceptualization, methodology, validation, and writing-original draft. XX was involved in conceptualization, methodology, validation, and writing-review and editing. All authors contributed to the article and approved the submitted version.

## Funding

Weihong Chen appreciates the National Natural Science Foundation Project of China (Grant No. 72174067) and the Key Research Base of Humanities and Social Sciences in Guangxi Universities and the project of Guangxi Development Strategy Institute (Grant No. 2022GDSIYB02).

## Conflict of interest

The authors declare that the research was conducted in the absence of any commercial or financial relationships that could be construed as a potential conflict of interest.

## Publisher’s note

All claims expressed in this article are solely those of the authors and do not necessarily represent those of their affiliated organizations, or those of the publisher, the editors and the reviewers. Any product that may be evaluated in this article, or claim that may be made by its manufacturer, is not guaranteed or endorsed by the publisher.
