# Supplementary material for: Top management team faultlines and corporate industrial diversification: The mediating role of strategic attentional breadth
Source: Front Psychol. 2023 Feb 23;14:1102192. doi: 10.3389/fpsyg.2023.1102192 (PMC9996410; doi:10.3389/fpsyg.2023.1102192)
Supplement: Supplementary file 1 [file Data_Sheet_1.docx]

**Appendix**

**TABLE 1 Industry distribution of sample enterprises**

| **Industry code** | **Industry name** | **Observation** | **Percentage (%)** |
| --- | --- | --- | --- |
| A | Agriculture, forestry, animal husbandry and fishery | 180 | 1.64 |
| B | Mining industry | 371 | 3.38 |
| C | Manufacturing industry | 6,372 | 58.08 |
| D | Industry of electric power, heat, gas and water production and supply | 373 | 3.4 |
| E | Construction industry | 507 | 4.62 |
| F | Wholesale and retail industry | 559 | 5.09 |
| G | Transport, storage and postal service industry | 235 | 2.14 |
| I | Industry of information transmission, software and information technology services | 1,079 | 9.83 |
| K | Real estate industry | 463 | 4.22 |
| L | Leasing and commercial service industry | 179 | 1.63 |
| M | Scientific research and technical service industry | 162 | 1.48 |
| N | Water conservancy, environment and public facility management industry | 197 | 1.8 |
| O | Industry of resident service, repair and other services | 14 | 0.13 |
| P | Education | 6 | 0.05 |
| Q | Health and social work | 32 | 0.29 |
| R | Industry of culture, sports and entertainment | 164 | 1.49 |
| S | Diversified industries | 79 | 0.72 |
|  | Total | 10,972 | 100 |

**TABLE 2 Categories and example dictionary words of strategic issues used to measure strategic attentional breadth**

| **Strategy categories** | **Number of words** | **Example dictionary words** |
| --- | --- | --- |
| 1. Alliance Partner Strategies (APS) | 27 | “合作”; “协议”; “合伙”; “联合”; “合资” |
| 2. Customer-Orientated Strategies (COS) | 22 | “客户”; “服务”; “用户”; “消费者”; “市场调研” |
| 3. External Stakeholder Management Strategies (STM) | 32 | “政策”; “股东”; “社会”; “投资者”; “媒体” |
| 4. Financial and Risk Management Strategies (FRM) | 69 | “利润”; “风险”; “资本”; “金融”; “负债” |
| 5. Internal Organizational Orientated Strategies (IOS) | 57 | “体系”; “人才”; “内部”; “组织”; “文化” |
| 6. Low Cost and Efficiency Strategies (LCE) | 33 | “费用”; “优化”; “降低成本”; “外包”; “经营效率” |
| 7. Mergers, Acquisitions, and Firm Scope Strategies (MAS) | 19 | “范围”; “合并”; “收购”; “融合”; “并购” |
| 8. New Market Entry Strategies (NME) | 45 | “扩大”; “进入”; “国际化”; “新业务”; “新兴产业” |
| 9. Product Marketing Strategies (PMS) | 27 | “品牌”; “媒体”; “广告”; “商标”; “市场调研” |
| 10. Resource and Capability Development Strategies (RCD) | 27 | “能力”; “资源”; “制造”; “研发”; “大数据” |
| 11. Social Strategies (SOC) | 37 | “环保”; “绿色”; “社会责任”; “社区”; “慈善” |
| 12. Product Innovation Strategies (PIN) | 44 | “产品”; “发展”; “研发”; “开发”; “创新” |
| 13. Business Model Innovation Strategies (BMI) | 15 | “平台; “新业务”; “经营模式’; “商业模式’; “业务模式” |

**Notes:** Strategy categories and dictionary words were modified based on Eklund and Mannor (2021).
